# Horsepox and the need for a new norm, more transparency, and stronger oversight for experiments that pose pandemic risks

**DOI:** 10.1371/journal.ppat.1007129

**Published:** 2018-10-04

**Authors:** Tom Inglesby

**Affiliations:** 1 Johns Hopkins Center for Health Security, Department of Environmental Health and Engineering, Johns Hopkins Bloomberg School of Public Health, Johns Hopkins University, Baltimore, Maryland, United States of America; 2 Department of Medicine, Johns Hopkins Univeristy School of Medicine, Baltimore, Maryland, United States of America; University of Pittsburgh, UNITED STATES

In January, Dr. David Evans from the University of Alberta and his colleagues published a scientific paper describing the synthesis of the horsepox virus de novo [1]. Horsepox doesn’t cause infection in humans, but another related virus—*Variola major*—causes smallpox. The publication of the horsepox synthesis process lowers technical hurdles for making smallpox de novo. This commentary argues that there are serious potential adverse implications of this work that don’t justify the purported benefits. It also makes the case that there should be a new norm related to experiments that increase pandemic risks and that there should be more transparency and stronger oversight for biological research and science that increases pandemic risks.

## Implications of synthesis

Horsepox is in the same viral family as smallpox; both are orthopox viruses. There has been no prior published report of an orthopox synthesis, so this experiment is the first time researchers have published a description of how to make a virus closely related to smallpox. In doing and publishing the horsepox synthesis work, the researchers have reduced uncertainties and addressed potential barriers that scientists would encounter in an effort to synthesize smallpox. In the paper, the scientists describe how they addressed “challenges” in the work [1]. For a scientific group determined to synthesize smallpox de novo, the paper would be useful. Drew Endy, a synthetic biologist on WHO’s Advisory Committee on Variola Virus Research (ACVVR), said about the publication, "There are things in this paper that I wouldn't know how to do and had never been done before" [2]. Other virologists have commented that they did not think the paper was a major technical advance; even if this were the case, it seems quite ill-advised to publish the full prescriptive details of the synthesis in one manuscript. Even Evans and colleagues said in the conclusion of their paper, “… This is clearly an example of dual-use research, and observations like these pose significant challenges for public health authorities” [1].

Why be concerned about making it easier to synthesize smallpox de novo? There are two primary reasons. First, there are intentional, extraordinary barriers in place to obtaining live samples of smallpox. There are only two official repositories of smallpox in the world (the United States Centers for Disease Control and Prevention [CDC] in Atlanta, Georgia, and the State Research Centre of Virology and Biotechnology [VECTOR] in Novosibirsk, Russia) [3], and to do research with smallpox in one of these facilities requires the permission of the ACVVR. No other pathogen has this highly restrictive global oversight approach in place. Synthesizing smallpox would be a way to circumvent these comprehensive restrictions to obtaining the smallpox virus.

The second reason for high concern is the potential severe impact of a new smallpox outbreak in our world today. Smallpox was declared eradicated from nature in 1980 after a 14-year global effort led by WHO and Dr. D. A. Henderson, who later founded what is now the Johns Hopkins Center for Health Security, where I serve as director [4]. Prior to its eradication, smallpox was infecting 10 to 15 million people each year, and it had killed an estimated 300 million in the 20th century alone [5]. In today’s world, virtually no one is immunized against smallpox. Governments have limited smallpox vaccine reserves, mostly concentrated in a limited number of countries. Were a smallpox epidemic to accidentally or deliberately start now, it could trigger a global catastrophe.

## Risks and benefits?

One of the proposed benefits of this work was that it would lead to a better smallpox vaccine. The case that this strain could be used to create a vaccine as effective and safer than the current smallpox vaccines is a hypothesis that could only be proven by substantial investment and many years of work. Moreover, there are no indicators that government or nongovernmental entities would be willing to provide the substantial funding that would be required over many years to complete that work [6]. Even in the case that there had been evidence that a better smallpox vaccine could have been developed with this strain and that there had been government interest in developing a new vaccine, a broader engagement with those concerned with the risks and those arguing for the benefits should have been undertaken. Perhaps that process would have resulted in identifying another path toward meeting the vaccine goals of the researchers without performing and synthesizing horsepox de novo. This now appears to be the case: the CDC has noted that it would have been willing to share its strain of horsepox, which would have obviated the need for horsepox synthesis [2].

Given the potential consequences of facilitating de novo orthopox synthesis, it would be reasonable to expect that this experiment had undergone some kind of extraordinary review that was commensurate with the risks entailed. It is not clear whether that was the case. Evans and colleagues report that they received approval from the University of Alberta Biosafety Officers and were in compliance with the Canadian Biosafety Standard published by Public Health Agency of Canada [1]. But Dr. Evans also believed that the “authorities, however, may not have fully appreciated the significance of, or potential need for, regulation or approval of any steps or services involved in the use of commercial companies performing commercial DNA synthesis, laboratory facilities, and the federal mail service to synthesize and replicate a virulent horse pathogen” [7].

The details have not been provided regarding what was conveyed to whom, what factors were considered, or how risks and benefits were weighed. While Dr. Evans was a member of WHO’s ACVVR, that committee’s approval was not sought, and the committee was not informed about the work until it was completed [2].

The greatest risks posed by this experiment were not solely or primarily biosafety in the traditional sense of protecting the laboratorians or the surrounding community of people or animals from infection following an accident. The greatest risks posed by this work arose from the information conveyed in the paper that makes it easier to create smallpox. That is not a biosafety problem confined to that institution and its surrounding community. That is potentially a global risk.

Publication of the synthesis process was not necessary to achieve the aim of studying it as a vaccine strain. Nonetheless, following the horsepox synthesis approval and then its completion, the researchers submitted the work for scientific publication. A number of concerned people from science and public health wrote letters to the publisher recommending it not be published given the risks outlined above [2]. In response, the publisher conveyed that the paper had been reviewed by its own internal advisory committee and that it would move ahead with publication. The deliberations of that committee have not been published, so it is not clear what that process entailed.

## Need for a new norm, more transparency, better oversight

Three changes should be pursued in order to address future experiments that could substantially increase pandemic risks: the adoption of a new norm in science related to this category of work, more transparency in the review of experiments that could increase pandemic risks, and increased oversight of this realm of work.

Firstly, scientists and the medical and public health community should embrace a new scientific norm. We should acknowledge that there are experimental efforts that could result in the creation of new pandemic risks, whether they are the creation of novel strains of pandemic potential, the publication of new simpler techniques to create eradicated pandemic viruses, or other means of increasing such risks. The other component of this new norm would be that experiments that fall into that category of potentially increasing pandemic risks should not be pursued without clear benefit, and if they are pursued, it should be with much more transparency, special review, and oversight.

One of the researchers’ stated benefits of the horsepox experiment was showing that this synthesis was possible [8]. This argument should not be an acceptable reason for doing this kind of work. That kind of argument could be used to justify many experiments that increase pandemic risks: a new pandemic risk would then be created in order to show a new pandemic risk exists. Instead, what this proposed experiment should have done is to trigger an extraordinary review and oversight process that considered benefits and risks.

Secondly, more transparency should be brought to the consideration of experiments that could substantially increase pandemic risks. Strong emphasis should be placed on transparency at the proposal design, approval, and funding stages. In this horsepox case, the scientists doing the work were aware of implications of the work that others involved in the review process may not have appreciated [7]. More transparency and external consultation at the design phase may have resulted in the CDC sharing a horsepox strain that could have been used for vaccine development purposes [2].

Although the emphasis on review and oversight for these kinds of risks should be at the proposal design phase, there will be experiments in which pandemic risks are only recognized later in the process. When something critical has been missed in the design, approval, and funding processes, a decision not to publish may be a necessary decision. For that reason, there should be more transparency at the publication review process for experiments that pose these kinds of risks. It would be valuable if the deliberations of the journal review process for the horsepox experiment were made public so that others working through these issues can learn from that process. There should also be expert technical and policy advice from the government, and a national advisory board should be created that could provide recommendations for journals having to make these decisions.

That said, hoping that the review process for a scientific journal will be the step that prevents the publication of science that increases pandemic risks is not a good strategy. Publication is very late in the process. The paper has been reviewed by many at that point. Each journal has its own review process, criteria, and advisors. Multiple papers might reject the work because of concerns, but it only requires one willing publication to publish it. The right time for oversight is before the experiment starts or is funded. To consider and handle these kinds of risks, scientific journals need more guidance from other realms of the scientific community, government, and policy makers, as some have argued [9], and as the recent National Academy of Sciences (NAS) report on this topic emphasizes [10].

Similarly, details of the institutional and regulatory review process for the horsepox experiment proposal have not been made available publicly, but they should be so that the community can learn from that process.

Finally, proposed experiments with the potential to increase or create new pandemic risks deserve special oversight and review before approval or funding. In the US, the Health and Human Services Framework for Guiding Funding Decisions about Proposed Research Involving Enhanced Potential Pandemic Pathogens (HHS P3CO Framework) requires a special review process for experiments that propose to create novel strains of pathogens with pandemic potential [11]. It is not a perfect policy in the sense that it has not been made clear to the scientific, medical, and public health community how broad the scope is, who does the review, how it is done, and how many other implementation issues are handled. As written, it is not clear whether it would have slowed or stopped the horsepox synthesis work. However, the HHS P3CO Framework is a step in the right direction, because it is written policy acknowledging there are experiments that carry the potential of extraordinary pandemic risks.

Other countries and scientific bodies should now engage with this problem and develop functioning policy that addresses it. Having countries adopt policies that oversee and govern science with the potential to increase pandemic risks would be an important step forward, even if those initial policies are imperfect. If more effective policies are developed on this issue elsewhere in the world, the US should consider adopting them. Ideally, different national policies on these issues should be aligned in ways that foster international scientific collaboration and publication given that science is an inherently international and collaborative field. The special review process used for experiments with pandemic risks should require the inclusion of scientists, public health and medical experts, ethicists, and others with the expertise needed to make impartial, nonconflicted judgments about the risks and benefits of proposed experiments that carry pandemic risks.

The experience of horsepox demonstrates that we do not yet have norms, transparency, or review and oversight systems in place to address experiments that increase pandemic risks. We should learn all that we can from the horsepox case. We should use it to help create the national and international systems that governments and scientific agencies will need to be able to address pandemic risks that emerge out of proposed experimental work in the life sciences in the time ahead.
